# T cell senescence: a new perspective on immunotherapy in lung cancer

**DOI:** 10.3389/fimmu.2024.1338680

**Published:** 2024-02-13

**Authors:** Mengge Huang, Yuetong Wang, Liguang Fang, Cun Liu, Fubin Feng, Lijuan Liu, Changgang Sun

**Affiliations:** ^1^ College of First Clinical Medicine, Shandong University of Traditional Chinese Medicine, Jinan, China; ^2^ College of Traditional Chinese Medicine, Weifang Medical University, Weifang, China; ^3^ Department of Oncology, Weifang Traditional Chinese Hospital, Weifang, China

**Keywords:** T cell senescence, immune senescence, immunotherapy, malignant lung tumor, T cell

## Abstract

T cell senescence is an indication of T cell dysfunction. The ability of senescent T cells to respond to cognate antigens is reduced and they are in the late stage of differentiation and proliferation; therefore, they cannot recognize and eliminate tumor cells in a timely and effective manner, leading to the formation of the suppressive tumor microenvironment. Establishing methods to reverse T cell senescence is particularly important for immunotherapy. Aging exacerbates profound changes in the immune system, leading to increased susceptibility to chronic, infectious, and autoimmune diseases. Patients with malignant lung tumors have impaired immune function with a high risk of recurrence, metastasis, and mortality. Immunotherapy based on PD-1, PD-L1, CTLA-4, and other immune checkpoints is promising for treating lung malignancies. However, T cell senescence can lead to low efficacy or unsuccessful treatment results in some immunotherapies. Efficiently blocking and reversing T cell senescence is a key goal of the enhancement of tumor immunotherapy. This study discusses the characteristics, mechanism, and expression of T cell senescence in malignant lung tumors and the treatment strategies.

## 1 Introduction

Maintaining a healthy intra- and intercellular environment in the human body requires the timely initiation of immune responses to recognize and eliminate pathogens and mutated malignant tumor cells. Aging causes dysregulation of the immune system, primarily in the form of immune senescence. T cell senescence is the main characteristic of immune senescence that includes the progressive loss and dysfunction of the immune system; the reduced ability of immune surveillance to inflammation, injury, and infection; the reduced ability to remove harmful elements (such as pathogenic microorganisms); and the failure of the immune system to promptly clear malignant tumor cells to prevent the survival and progression of tumor cells. The immune function of patients with malignant lung tumors is impaired, and there is a high risk of recurrence, metastasis, and mortality. Immune checkpoint inhibitor and Chimeric Antigen Receptor T-Cell (CAR-T) immunotherapy is promising for treating solid tumors and some hematological malignancies. Meanwhile, combination vaccine and CAR-T therapies can effectively stimulate endogenous systems to avoid evasion of antigen-negative tumors. T cells are a core component of the immune system in the human body (1). However, the presence of senescent T cells leads to the ineffective clearance of tumor cells and immunosuppression in the tumor microenvironment (TME), which negatively affects the efficacy of immunotherapy to some extent (2).

Chronic systemic inflammation increases with age; therefore, older adults express more inflammation-related genes (3). In a series of reactions, the pathophysiological characteristics of a lung can trigger the upregulation of pro-inflammatory and repelling factors, causing chronic lung inflammation and promoting the development of lung cancer, which induces premature aging, also known as inflammaging (4).

Lung cancer treatment has undergone a significant transformation over the past decade, and our understanding of lung cancer biology in the macro- and microenvironment has led to more opportunities for developing immunotherapy applications (5). T cell senescence exists in malignant tumors and is an important indication of T cell dysfunction, which affects the occurrence and development of malignant tumors. T-cell functional status is a key determinant of effective anti-tumor immunity and immunotherapy, and T-cell senescence is a common feature of cancer progression, with T-cell senescence leading to a reduced ability to kill cancer cells. If a patient’s T-cell functional status is abnormal or senescent, then the cancer will continue to develop. Therefore, it is important to study the association role of T cell senescence in lung cancer to improve the efficacy of immunotherapy and the prognosis of lung cancer. This review discusses the characteristics and mechanism of T cell senescence, its expression in malignant lung tumors, and therapeutic strategies.

## 2 T cell senescence

### 2.1 T cell senescence characteristics

Senescent T cells exhibit the same characteristics as senescent cells. First morphologically senescent T cells are flatter and larger. Senescent T cells have shown high expression of age-related β-galactosidase (SA-β-gal), telomere shortening, loss of telomerase activity and genomic instability, and constant DNA damage in cells by external environmental factors and internal biological processes (6). The widespread functional inactivation of p53 and RB results in G1/S checkpoint defects of the cell cycle and continuous accumulation of DNA damage (7), causing cell cycle arrest, resulting in decreased cell proliferation. In addition, exhausted T cells were characterized by high expression of CD45RA and C-C chemokine receptor type 7 (CCR7), and naive cells turn into effector memory T cells (8) CD27/CD28 costimulatory molecules on the surface of T cells are consistently low in expression. CD27 is an important molecule for T cells to maintain immunity, CD28 is an important second signal in T cell activation, and loss of CD27 and CD28 will lead to reduced immune capacity (9). Furthermore, senescent T cells highly express molecular markers, such as killer cell lectin-like receptor subfamily G member 1 (KLRG1) and CD57. KLRG1 is associated with signal transduction, and CD57 is associated with T-cell proliferation damage (10). Senescent T cells exhibit senescence-related secretory phenotypes; even if they cannot proliferate, they can still produce and secrete many pro-inflammatory factors after stimulation and activation (11). Interleukin (IL) 2, IL-6, IL-8, interferon-γ (IFN-γ), and transforming growth factors (TGF), such as IL-10 (12), are inflammatory mediators that further promote the formation of TME. It is still controversial as to whether senescent T cells express exhaustion markers such as PD-1 as well as LAG-3 (13). **Table 1** shows the molecular features and markers of T cell senescence.

**Table 1:** Markers of T cell senescence.

Category	Markers	Refs
SASP	IL-2、IL-6、IL-8, TNF-α、TNF-γ、Granzyme ↑	(14–17)
SA-β-gal	Senescent cell biomarkers	(18)
Cell cycle arrest	P53, p21, p16↑	(11, 19)
DNA damage	ATM	(1)
Metabolic changes	ROS↑	(20)
phenotypic markers	CD28,CD27↓ CCR7,CD45RO↓ CD45RA↑ CD57,CD95↑	(8, 9)
	KLRG-1↑	

"↑" for high expression, "↓" for low expression.

### 2.2 T cell senescence and T cell exhaustion

**Figure 1:**
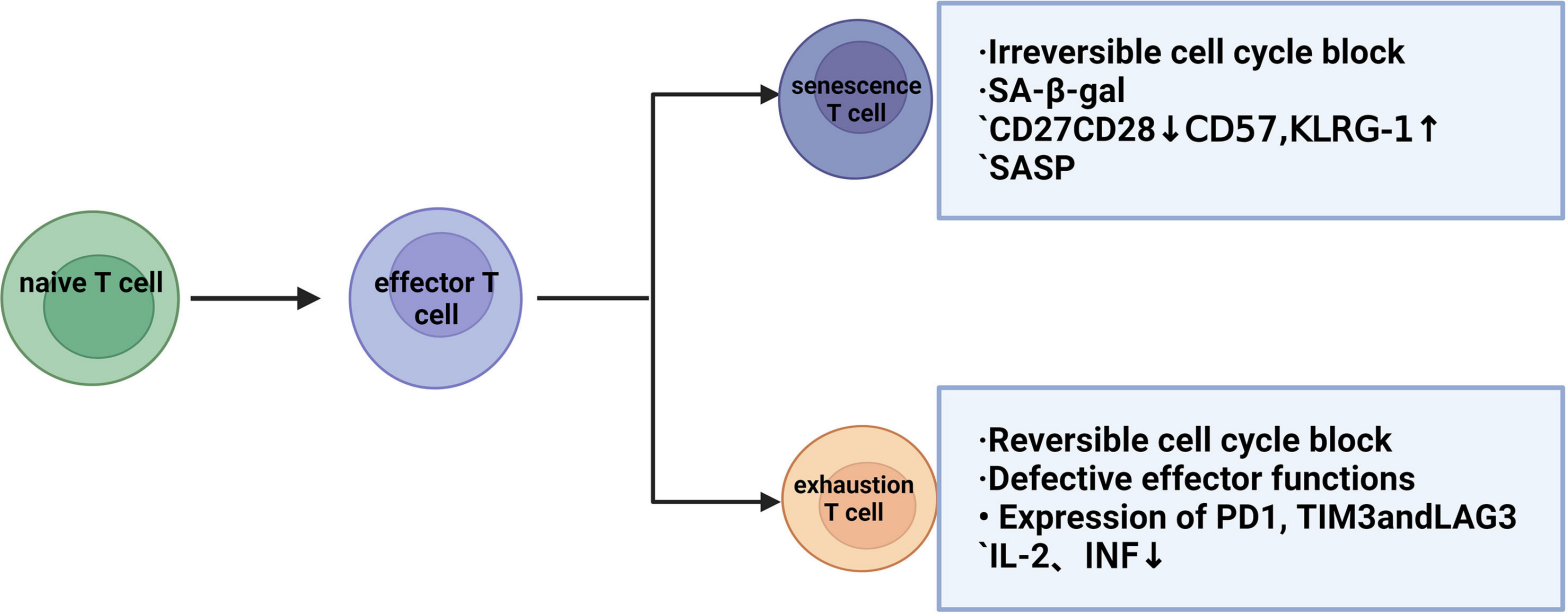
Differences between T-cell senescence and T-cell exhaustion.

T-cell exhaustion indicates a state of cellular dysfunction in the immune system. For tumor cells, the immune cells that play a major role are CD8+ T cells, and due to the continuous stimulation of chronic inflammation, the killing function of T cells will gradually decline and show a state of exhaustion. Exhausted and senescent T cells have a functional deficiency in developing tumor immunity (21) but exhibit different molecular phenotypes and functions (22), which progress the tumor and prohibit any anti-tumor function. IFNα-treated cells (the factor that drives T-cell differentiation to end effectors) also result in p38 activation, which may explain the low proliferative capacity of the end effector cell population, as well as the similarity to the phenotype and function of senescent T cells. Thus, although exhaustion and senescence are two distinct processes, they are often confused. Assessment of T cell function (proliferation, cytokine secretion, and cytotoxicity) by evaluating T cells is important to distinguish whether T cells are exhausted or senescent. T-cell exhaustion is a protective mechanism against excessive immune damage to normal tissues in the tumor microenvironment and is a pathological state caused by the autoimmune system repairing long-term viral or bacterial infections or chronic tissue damage. In these cases, perhaps (partial) T-cell exhaustion strikes a balance between maintaining limited infection control and modulating immunopathology (23). Senescent T cells have a senescence-related secretion phenotype (SASP), which can produce and secrete many pro-inflammatory factors after stimulation and activation, even if they cannot proliferate (11). In exhausted T cells, IL-2 production, tumor necrosis factor, and proliferative capacity are decreased, and in the final stage, T cell effector function is completely lost. This again shows that senescent and exhausted T cells are in two different states (24, 25). Concurrently, T cell exhaustion is accompanied by the expression of inhibitory surface receptors, including PD1, CD160, 2B4, lag3, and CTLA-4. Thus, poor effector function and high expression of inhibitory receptors are key features of T cell exhaustion (23). Current clinical trials using the immune checkpoint CTLA-4 and/or PD-1/programmed cell death ligand 1 (PD-L1) have shown promising efficacy in patients with malignant tumors (26). However, the benefit is still limited, suggesting that T cell exhaustion is not entirely responsible for the impaired anti-tumor function. T-cell senescence and T-cell exhaustion can be assessed in terms of proliferation, cytokine secretion, and cytotoxicity, at the same time, when the body is subjected to chronic infections or produces tumors, the constant antigenic stimulation leads the T-cells towards exhaustion/senescence. T-cell responses to infection and tumors depend on epigenetic remodeling induced by metabolic reprogramming and synergistic interactions between immune cells. In particular, T cell effects and memory differentiation, exhaustion, and senescence are closely regulated by the metabolic-epigenetic axis (27) (**Figure 1**).

### 2.3 T cell senescence mechanism

Physiological and pathological mechanisms cause T cell aging. T lymphocytes age during the physiological process of immune aging along with the gradual degeneration of the thymus gland (28). Additionally, the activation of age-related pathways, telomere shortening, mitochondrial dysfunction, inflammatory stimulation, and genomic instability contribute to pathological aging (29).

#### 2.3.1 Activation of age-related pathways

Mitogen-activated protein kinase (MAPK), a class of serine/threonine protein kinases, plays an important role in cell proliferation, differentiation, transformation, and apoptosis. Moreover, the MAPK signaling pathway is a key signaling pathway for T cell aging. Cyclic adenosine monophosphate (cAMP) and other cyclic DNA damage can activate the MAPK signaling pathway. Furthermore, cell cycle regulatory molecules, such as P53, P21, and P16, are activated by P38MAPK to cause cell cycle arrest, thus preventing DNA replication (30). Converting growth factor β-activated kinase-binding protein 1 to a complex with AMP-activated protein kinase (AMPK) also activates p38MAPK, which initiates endogenous DNA damage response, leading to T cell senescence through recruitment of ataxia telangiectasia mutated (ATM) (1). In senescent CD4+ T cells, sestrin protein (SESN1/2/3) promotes senescence by inhibiting mTOR through the sMAC complex and activating AMPK to upregulate P38, Erk, and JNK (31). The p38MAPK and ERK1/2 signaling pathways in T cells induce T cell senescence through the activation of Tregs, and Treg cells cooperate with MAPK, STAT1, and STAT3 signals to control T cell senescence. Therefore, inhibition of the T cell MAPK signaling pathway can prevent the Treg cell-mediated T cell aging process (32).

#### 2.3.2 Telomere shortening

Studies have shown that when the telomeres in immune cells are shortened, a variety of immune cells, including T lymphocytes, will exhibit senescent and apoptosis phenotypes, affecting cell function and activity and further affecting the stability of the body’s immune system (33). Shi et al. found that T cell senescence is related to telomeres, and the defective expression of the shelterin protein complex will affect the formation of the telomere T-loops in cells. This results in the loss of a “cap structure” at the end of chromosomes, destabilizing the genome at the end of chromosomes and increasing cell apoptosis or aging. Partial subunit expression defects of the shelterin complex of CD4^+^T cells in patients with BD suggest abnormal telomere status, loss of the original telomere protection of chromosomes, vulnerability to DNA damage and other attacks, and acceleration of cell aging and apoptosis (34). NF-kB signaling and exposure to inflammatory molecules, such as IL-6 or TNF, can regulate telomere length and activity (35). Studies have shown that telomere shortening and abnormal telomerase activity are closely related to autoimmune diseases like rheumatoid arthritis and systemic lupus erythematosus (36–39). T cell subpopulations exhibit age-related reductions in telomere length, especially in chronic infectious diseases, such as cytomegalovirus (CMV). One of the most common changes in human short telomere syndrome is idiopathic pulmonary fibrosis (IPF), in which patients who have undergone lung transplantation have impaired immunity to CMV, demonstrating that T cell function is subsequently reduced after telomere shortening.

Although different studies have yielded inconsistent results, telomere shortening generally suggests a worse prognosis for lung cancer patients (40). Shortened peripheral blood telomere length was found to be associated with an increased risk of death in cancer patients (41). Weischer (42) found that in a Danish population, reduced telomere length detected before a lung cancer diagnosis was associated with increased mortality in patients with lung cancer. Kim (43) found that excessive telomere length measured in patients with early non-small cell lung cancer (NSCLC) was associated with an increased risk of recurrence after radical lung cancer resection. To clarify the impact of telomere shortening on the survival of patients with early NSCLC, Jeon (44) used quantitative polymerase chain reaction to measure the relative telomere length in tumor tissues of 164 patients with surgically removed NSCLC and the association between telomere length and overall survival (OS) and disease-free survival (DFS) were analyzed. Compared with older patients, the healthy older adult population had longer telomeres, higher telomerase activity, and stronger cell proliferation ability. Telomere length is shortened in most tumors, including in older patients with lung cancer. Changes in telomere length are associated with the risk of lung cancer and may be used as a prognostic indicator of lung cancer, especially in NSCLC (45).

#### 2.3.3 Mitochondrial dysfunction

Mitochondrial dysfunction is another mechanism of T cell senescence that occurs in most tissues and cell types, including T cells (46). In the tumor microenvironment, cytotoxic T cells are activated by tumor antigens through T cell receptor (TCR) signaling, resulting in a durable and effective anti-tumor immune response. T cells differentiate from initial state T cells to effector T cells upon receiving antigen activation, at which point the expression levels of specific genes involved in aerobic glycolysis and regulation of effector functions will increase. After successful clearance of antigen by the body, memory T cells utilize fatty acid oxidation (FAO) and oxidative phosphorylation (OXPHOS) to satisfy energy requirements and long-term survival properties (27). During abnormal cell metabolism, mitochondrial function is impaired; biosynthesis capacity, T cell activation, and functional persistence are reduced immunosuppressive cells, such as Treg cells, compete with T cells for glucose, triggering ATM-related DNA damage and T cell senescence (47). Mitochondria are the main sources of intracellular reactive oxygen species (ROS), which are important in regulating cellular life activities, such as oxidative stress, apoptosis, and gene expression (48). Mouse bone marrow mesenchymal stem cells were cultured *in vitro* by Li et al., who found that C1q/TNF-associated protein 9 activates the peroxisome proliferator-activated receptor-γ coactivator 1α (PGC-1α)/AMPK signaling pathway, enhancing the antioxidant capacity of cells. However, silencing of the PGC-1α or AMPK gene can increase the intracellular ROS level, resulting in the decrease of mitochondrial respiration ability and ATP production and then cell senescence (20). Ye (49) found that tumor-derived endogenous cAMP caused T cell DNA damage and induced T cell senescence. Activation of Toll-like receptor 8 (TLR8) in tumor cells can down-regulate the level of tumor-derived cAMP, block and reverse the aging process, and restore the anti-tumor ability of T cells. Therefore, understanding the molecular mechanism of T-cell senescence can provide a new vision for immunotherapy to some extent.

#### 2.3.4 Inflammatory stimulation

Immune senescence and inflammation interact and persist throughout life. If the body is constantly exposed to repeated stimulation of tumor antigens, it will cause DNA damage, telomere wear, and the aging of immune cells. However, the aging of the immune system will cause changes in the structure and function of immune cells. Numerous inflammatory cytokines are continuously secreted during the aging of immune cells, resulting in long-term sustained inflammatory damage to the body. During infection, toxicity, and irritation conditions, there are high concentrations of inflammatory factors in serum, such as IL-6, IL-8, TNF, C-reactive protein (CRP), soluble glycoprotein 130 (SGP130, involved in IL-6 signal transduction, sCD30, and MCP-1). Senescent T cells secrete the SASP phenotype, and SASP factors affect neighboring cells or promote angiogenesis, progressing tumor cell growth, invasion, and metastasis. Senescent T cells secrete the SASP phenotype, and SASP factors affect neighboring cells or promote angiogenesis, progressing tumor cell growth, invasion, and metastasis. The senescence-associated secretory phenotype (SASP) is an effector arm of senescence, leading to senescent cell clearance or chronic inflammation, tumor suppression, and tumor promotion, among others. Tumor-promoting SASP effects are associated with pro-inflammatory SASP factors (e.g., IL-6 and IL-8), which promote epithelial-to-mesenchymal transition, recruitment of tumor-promoting macrophages, and suppression of cytotoxic T cell function (50). However, inflammatory responses may also contribute to inflammatory aging by accelerating telomere length shortening. Meanwhile, DNA damage, DNA double-strand breaks, DNA damage repair (DDR) defects, telomere shortening, and reduced telomerase activity are common in aging T cells. In this process, DNA-dependent protein kinase breakdown subunits (DNA-PKCs) are activated by long-term DNA damage (51). DNA-PKCs activate NF-KB via intracellular signaling pathways, promoting the production of IL-1, IL-6, tumor necrosis factor, and other inflammatory factors, leading to further damage. Aging involves the degeneration of the thymus in the interactive process of immune and inflammatory senescence. Thymus degeneration exhibits tissue structure destruction and decreased thymus mass and cell number, leading to decreased initial T cell output and peripheral TCR pathogen detection. This results in the continued accumulation of senile cells, inflammatory senescence in the body, and increased SASP secretion. However, in the process of thymus degeneration, the decline of thymus function leads to the impairment of the negative selection function of T cells, reducing the acquisition of the T cell central immune tolerance mechanism. The T cell bank entering the peripheral lymphatic organs contains T cells which target their organs and tissues, causing autoinflammation and immune response; this is also an important factor in inflammatory aging (52).

## 3 The role of senescent T cells in the genesis and development of malignant tumors

**Figure 2:**
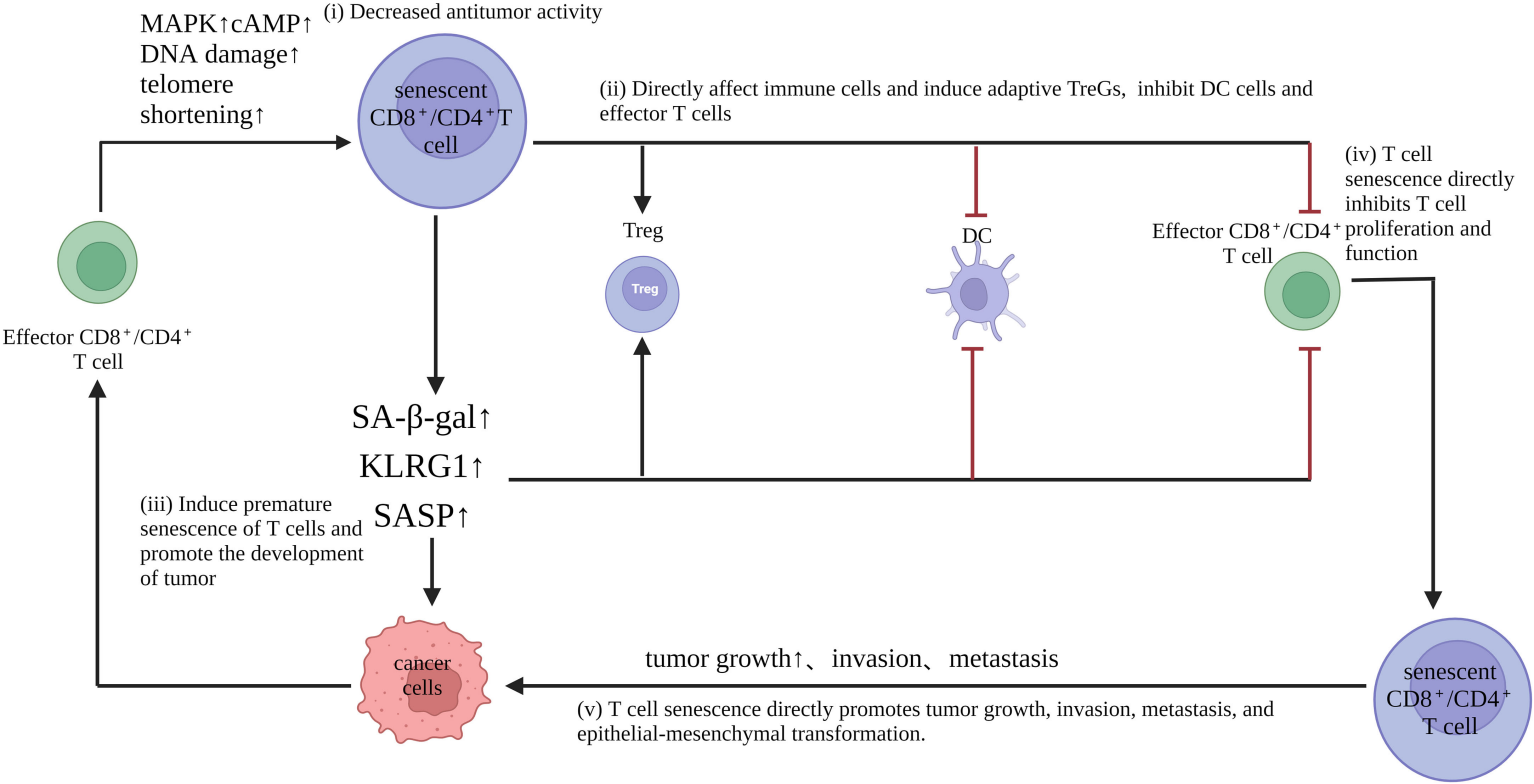
Role of senescent T cells in tumorigenesis and development: (i) Senescent T cells have impaired anti-tumor activity. Moreover, down-regulation of the costimulatory molecules CD27 and CD28, as well as the effector molecules perforin and granzyme, reduces proliferation, promotes cell cycle arrest and inhibits the expression of proliferative molecules. (ii) senescent T cells directly affect immune cells and induce adaptive Tregs, but inhibit DC and effector T cells (iii) senescent T cells exhibit an aging-associated secretory phenotype, which produces abundant pro-inflammatory factors, such as interleukin (IL)-2, IL-6, IL-8, and interferon-γ, which interfere with normal cell differentiation, although they are leukin cells that are leukocyte leukin, which promotes the occurrence of malignant tumors; (iv) T cell senescence directly inhibits T cell proliferation and function; (v) T cell senescence directly promotes tumor growth, invasion, metastasis, and epithelial-mesenchymal transformation.

Increasing evidence shows that aging T cells are important amplifiers and key immunosuppression agents in the inhibitory TME (49, 53, 54). Understanding the role of senescent T cells in tumor immunity opens the way for new immunotherapies. In addition to regulating the tumor microenvironment through SASPs, senescent T cells also act as a unique regulatory T cell subpopulation that can directly inhibit proliferative and activating functions and reduce pro-inflammatory cytokine secretion while inducing apoptosis of activated T cells *in vitro*. At the same time, senescent T cells with a suppressive role in the tumor microenvironment are able to promote the production of pro-inflammatory factors (TNF, IL-1b, and IL-6) and angiogenic factor, vascular endothelial growth factor A (VEGF-A) by monocytes/macrophages, which leads to renal tubulogenesis and tumor cell survival (2). The above evidence suggests that senescent T cells promote immune escape from tumors to some extent. Pretreatment levels of senescent T cells in peripheral blood correlate with tumor progression and prognosis, as well as with the prognosis of patients treated with chemotherapy. In advanced NSCLC, higher levels of senescent T cells independently predicted unfavorable OS and post-diagnostic progression-free survival (PFS), and patients with higher levels of senescent T cells in the peripheral blood had lower levels of IFN-g and higher levels of IL-6, which may partly explain why senescent T cells predicted poor chemotherapy efficacy (55, 56) Senescent cells produce a complex mixture of SASPs. In addition to this, inflammatory and immunomodulatory factors as well as repellency factors are significantly upregulated in senescent T cells (57). Meanwhile SASP factors, such as IL-6 and TNF-α promote T cell senescence in autocrine or paracrine forms, such as the CCL5/CCR5 axis that promotes tumor growth and migration, facilitates neovascularization, and induces immunosuppressive polarization of monocytes and myeloid-like cells, leading to the generation of M2-type tumor-associated macrophages and myeloid-derived suppressor cells (MDSCs) (58). However, whether senescent T cells play a positive or negative role in different tumors and at different stages of tumor development still needs to be further investigated (**Figure 2**, **Table 2**).

**Table 2:** T cell dysfunction in tumor.

T cell dysfunction in tumor		Ref
Inhibitory receptors	PD-1、CTLA-4、Tim-3、LAG-3	(59, 60)
Transcriptional regulation	T-bet、Eomes、Foxo1、Blimp-1、NFAT、TOX	(59)
Inhibitory cells	Treg cells、TAMs、cancer-associated fibroblasts and adipocytes endothelial cells	(59, 61)
Suppressive soluble mediators	IL-10、VEGF-A、TGF-β、IL-35	(59, 62)

### 3.1 T cell senescence and lung malignancy

*In vivo*, anti-tumor immunity, cellular immunity is the main way of anti-tumor immunity, and T lymphocytes play a key role in this process. The immune function of the elderly population also gradually decreases with age, which is what we call the key problem of T lymphocyte senescence, that is, immune senescence. From the perspective of immunology, the occurrence and development of tumors are closely related to the body’s immune state, which is mainly caused by the immune escape mechanism and the anti-tumor function of the immune surveillance system (63). Studies have shown that T-cell senescence is found in hematological malignancies (64–66).

Lung cancer is based on the aging of the body during its onset when complex immune remodeling occurs in the immune system (63, 67). The function of T cells has undergone important changes, namely, T lymphocyte senescence: the imbalance of T lymphocyte subsets in older patients and decreased ability to activate apoptosis and proliferative response to antigen and mitogen stimulation. In addition, it is noteworthy that T cells originating from healthy older individuals also exhibit this dysfunction (68). Additionally, the function of T cells depends on the combined action of various helper molecules on the membrane surface (69). Studies have shown that the expression of CD28 in the peripheral blood of patients with malignant lung tumors is significantly decreased (70), which indicates that the abnormal costimulatory pathway leads to the decrease of immune function in the body and is closely related to the occurrence and development of tumors. Activated efficient T lymphocytes were cleared by the Fas/FasL-mediated apoptosis pathway (71), and the expression level of the Fas (CD95) antigen on peripheral blood T cells of patients with lung malignant tumors was significantly higher than that of healthy individuals (72). Therefore, the abnormal regulation of Fas-mediated apoptosis of T cells may be related to the occurrence and development of malignant tumors. The Fas/FasL pathway can be a basis for exploring the intrinsic relationship and possible mechanism between T cell senescence and lung cancer, and provide the experimental basis and scientific strategy for immunotherapy of lung cancer in the elderly.

### 3.2 Regulation of T cells and their CD28/CD95molecules in the aging process of the body

Currently, lung cancer and digestive system tumors are the main types of cancer among the elderly in China. Aging is an inevitable stage of the body’s metabolic process, and the function of the immune system declines with the aging of the body, which is manifested in the gradual decline of the immune system’s response to antigens, thus leading to an increased susceptibility of the body to infectious diseases, tumors and autoimmune diseases.CD28 is a member of the immunoglobulin superfamily (IGSF), which is a homologous dimer glycoprotein with a relative molecular weight of 44KDa and is expressed on 95% of resting CD4^+^ and nearly 50% of resting CD8^+^ in human peripheral blood. As a costimulatory molecule expressed on the surface of T lymphocytes, CD28 binds to the B7 molecule on antigen-presenting cells (APC), thereby mediating the activation of T cells, promoting T cell proliferation and secretion of various cytokines, and providing survival signals for preventing T cells aging and apoptosis (73–75). Additionally, CD28 mediates the adhesion of T and B cells and prevents the clone from being unresponsive. CD28 costimulatory molecules on the naïve T cells provide a costimulatory signal to T cells that receive simultaneous TCR stimulation, inducing the expression of the IL-2R (interleukin-2) receptor and transcription of IL-2 genes on the T cell surface (76). The combination of the B7 molecule and CD28-activated Th1 and Th2 cells induced the proliferation and activation of Th1 cells, promoted the recovery of immune function, and activated anti-tumor immune response by Th1 cytokines (77). The aging immunity study centered on T lymphocytes found that CD28 in the peripheral blood of healthy individuals was downregulated with aging, and the activation and proliferation ability of T cells decreased correspondingly, suggesting that the decrease of immune function caused by an abnormal costimulatory signaling pathway was related to the occurrence and development of malignant tumors, and became one of the important characteristics of T cell senescence (78). CD95 (Fas/Apo-1) is mainly distributed on the surface of human-activated T and B lymphocytes and is an important apoptosis molecule, also known as the “death receptor,” belonging to the tumor growth and nerve growth factor receptor superfamily members. Its ligand FasL (CD178) is a member of the TNF ligand superfamily and transmits apoptotic signals to cells and induces apoptosis. The interaction of Fas and FasL can cause numerous antigen-activated T cells to initiate a caspase cascade, thereby inducing apoptosis, also known as activation-induced apoptosis (AICD) (79). However, cytotoxic T lymphocytes (CTL) and natural killer cells (NK) express TNF-α and FasL (80) to activate cell death programs, leading to apoptosis of target cells. Immunosenescence studies have found that the expression level of CD95 in human peripheral blood T cells increases with aging, which leads to senescent T cells, especially CD8^+^T cells, becoming more sensitive to TNF-α-mediated apoptosis signals (81), and the survival rate of T cells after activation decreases. Experimental results (82) also showed that the expression rate of the CD28 and CD95 antigen proteins in T cell subsets decreased and increased with aging, respectively.

### 3.3 The regulatory role of T cells and their CD28/CD95 molecules in anti-tumor immunity

It is well known that CD8^+^ cytotoxic T cells can only be activated into antigen-specific effector T cells with the stimulation of the APC surface antigen polypeptide—MHC-I molecular complex—therefore, specifically target cells to kill. Effective activation of T cells is a key step in the active immune process of the body against tumor cells (83). CD28, a T-cell-specific costimulatory molecule, is essential for TCR-mediated antigen recognition and signaling output. CD8^+^T cells can be divided into CD8^+^CD28^+^ T cells and CD8^+^CD28^-^ T cells (84) based on the costimulatory molecule CD28 is expressed or not, respectively. CD8^+^CD28^+^ specific killer cells differentiate into effector killer cells by cytokines secreted by CD4^+^Th cells. Cytotoxic substances, such as perforin and granzyme (85), are released to dissolve target cells or release cytokines to induce apoptosis of target cells to kill tumor cells specifically. Since CD4^+^T cells belong to MHC-II restricted T cells and cannot directly recognize tumor cells, they activate CTL by secreting IFN-γ and IL-2 when stimulated by APC antigen presentation; therefore, the two types of cells cooperate to kill tumors (86). Zou (87) showed that serum CD3^+^, CD4^+^, CD8^+^, and CD28^+^ T cells in older patients with lung cancer were significantly decreased compared with healthy older patients, and the CD28 mRNA content in peripheral blood of older patients with primary NSCLC was significantly lower than that in the healthy and non-cancer older groups. The abnormal decrease of CD28 mRNA content suggests that the body lacks an effective second signal to activate CD8^+^CTL cells, which allows the tumor to evade the host immune surveillance and tumor immune escape.

Fas/FasL pathways evade the human immune system from killing tumors. FasL expression in tumor cells promotes increased apoptosis of T cells activated by Fas high expression (88), resulting in immune escape. However, tumor cells could overexpress the anti-apoptotic gene product (bcl-2) (89) or not express Fas and Fas-related signal transduction molecules to resist the apoptosis mediated by the Fas/FasL pathway and avoid the killing action of activated CTL. The abnormal regulation of the Fas/FasL pathway leads to the inhibition of apoptosis and allows tumor cells to evade immune surveillance (90). Hoser (91) the expression level of Fas antigen on peripheral blood T cells of lung cancer patients was significantly higher than that of healthy people, I ndicating that abnormal Fas-mediated apoptosis of T cells may be related to the occurrence and development of malignant tumors. The experiment showed that CD95 was mainly increased in peripheral blood T cell subsets of older patients with NSCLC, and the CD95 mRNA content was significantly higher than that of the healthy and non-cancer group older patient groups. The Fas/FasL apoptosis pathway induces excessive apoptosis of T cells and their subsets, thus weakening the immune surveillance and attack ability towards tumor cells, leading to further proliferation and invasion of lung cancer cells (92).

## 4 Therapeutic strategies of T cells in lung malignancies

**Figure 3:**
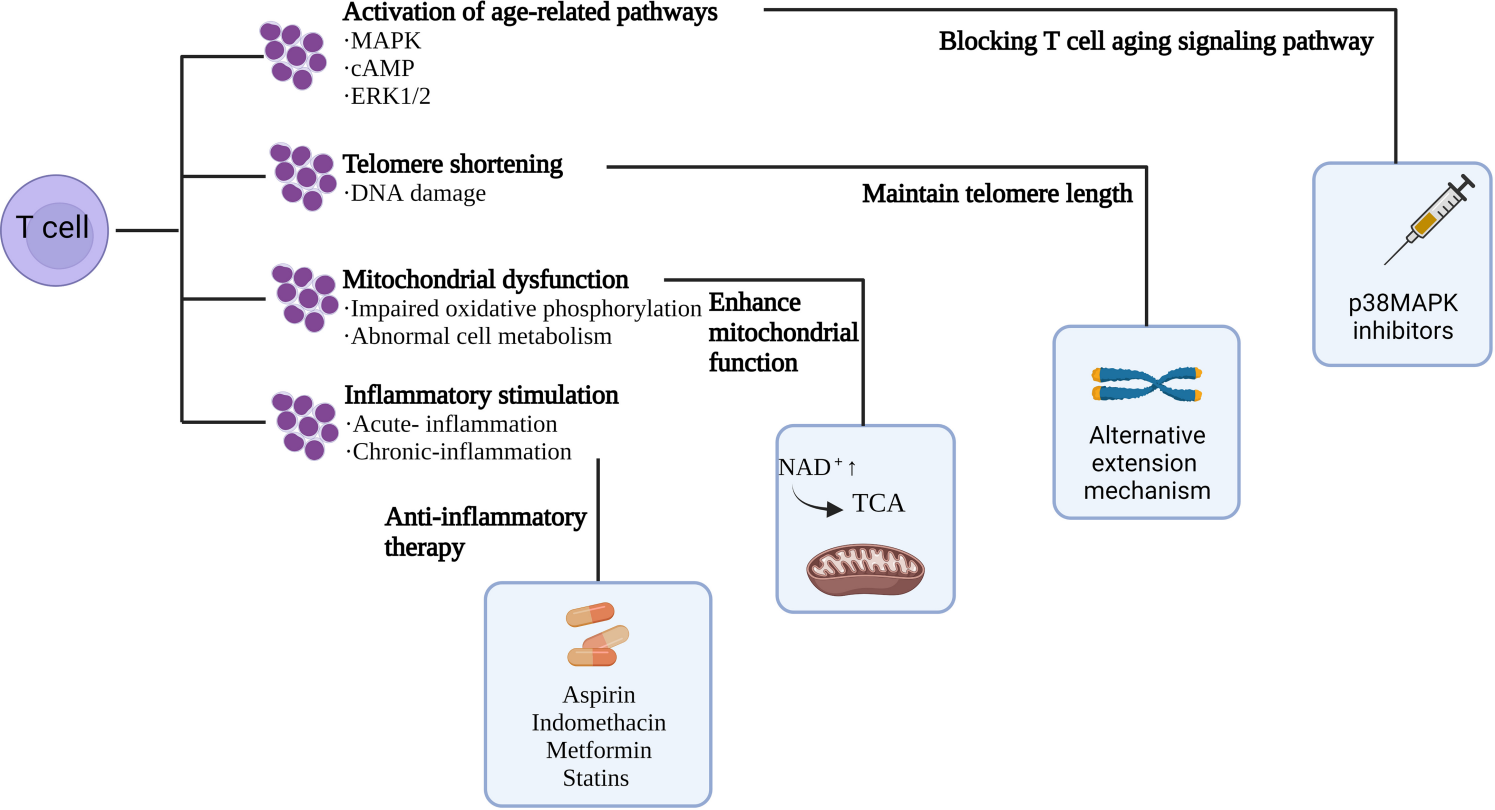
Mechanisms of T cell senescence and ways to reverse T cell senescence.

According to preclinical experiments, inhibitors targeting p38, ERK, JNK, and STAT signaling pathways prevented T cell senescence (93); and rapamycin, a mammalian rapamycin complex 1 (mTORC1) inhibitor, metformin, and BIRB 796, a p38 inhibitor, increase mitochondrial biogenesis and viability, and work by regulating metabolism to reverse T cell senescence (93–95). Another study demonstrated that a hypoxic environment caused by the accumulation of adenosine and cAMP induces T-cell senescence (49) can be treated with hyperbaric oxygen therapy to increase the chromosome length of peripheral blood cells and thereby reduce the number of senescent T cells (96). In addition to this, numerous studies have shown that NK cells, as the central participant in the immune surveillance of senescent cells, are able to rapidly capture abnormalities such as senescence. T-cell senescence was significantly ameliorated by infusing back NK cells, with a significant reduction in peripheral senescent and failing T cells in the infused subjects, and the T cells exhibited enhanced cytotoxicity (97). All evidence suggests that T cell senescence weakens anti-tumor capabilities as a strategy to evade immune surveillance. Therefore, developing strategies to reverse T cell aging and combat tumor immunity is important (**Figure 3**).

### 4.1 Blocking T cell aging signaling pathway

The key signaling pathway controlling the induction of T cell senescence is an important target to enhance anti-tumor immunity effectively. Fibroblasts can reverse growth arrest by blocking key cell cycles in early senescence (19, 84), which also provides key ideas for reversing T cell senescence. p38MAPK is the core target to reverse T cell senescence. Inhibition of p38MAPK in T cells reverses the process of DNA damage-related T cell senescence. The safety of the p38MAPK inhibitor has been confirmed in myelodysplastic syndrome and MM (98–100). Meanwhile, studies have shown that inhibition of the MAPK signaling pathway (101) can restore the cell cycle activity and proliferation ability of T cells. MAPK inhibitors have been widely used to treat patients with melanoma, and have been shown to enhance T-cell recognition of melanoma without affecting lymphocyte function (102, 103). Another method is by activating the TLR8 signaling pathway in Treg and tumor cells. cAMP inhibitors also inhibit tumor-induced T-cell senescence by reactivating TLR8 signaling (2). TLR8 agonists reduce cAMP production in tumor cells and inhibit glycolysis of tumor-derived Tregs without interfering with effector T cell metabolism (49), thereby preventing T cell senescence.

### 4.2 Enhance mitochondrial function

Decreased metabolic function and mitochondrial dysfunction are apparent in T cell senescence. Iron is an essential nutrient involved in cell metabolism, and its deficiency can lead to impaired cell function, anemia, nervous system damage, cancer, and loss of cell function. Jovian et al. incubated cells with different concentrations of FeSO_4_ in the medium and performed growth analysis at different time points (16, 24, and 48 h). Approximately 24 h after incubation with FeSO_4_ concentration, cell growth reached saturation, and the chronological lifespan (CLS) of cells incubated with different concentrations of FeSO_4_ was measured. The results showed that adding different concentrations of FeSO_4_ in the medium could prolong the CLS of yeast. The researchers also tested using different concentrations of FeCl_3_ and found results similar to FeSO_4_. These results confirm that iron supplementation can delay aging and prolong life by enhancing mitochondrial function (104). Mitochondria serve as the cellular centers for iron utilization and metabolism, and iron is a cofactor for several mitochondrial proteins, including iron-sulfur clusters and heme-containing proteins. Studies have shown that iron supplementation can significantly induce the expression of mitochondrial tricarboxylic acid (TCA) cycling genes. The TCA cycle metabolites alpha-ketoglutaric acid and oxaloacetic acid produce glutamic and aspartic acid through catalytic reactions in mitochondria, respectively. These results suggest that iron supplementation promotes TCA circulation by placing cells in a metabolic state conducive to regeneration. Moreover, mitochondria produce energy, participate in aerobic respiration, provide energy for cells, and are involved in cell differentiation, information transmission, and apoptosis. Nicotinamide adenine dinucleotide (NAD^+^), a key molecule in the process of mitochondrial energy production, decreases with the increase of age. Recently, experiments have demonstrated a new method to promote the synthesis of NAD^+^ by inhibiting 2-amino-3-carboxylic muconic acid 6-hemaldehyde decarboxylase (ACMSD). Increased levels of NAD^+^ enhanced mitochondrial function in Cryptothritis worms and mice (105). In a study conducted by Tineke van de Weijer et al., 21 patients with type 2 diabetes aged 57.7 ± 1.1 years were treated with a placebo or acipimox (250 mg/d oral, three times daily) for 2 weeks and found a substantial increase in ATP levels in muscle biopsies of patients treated with acipimox. Subsequently, it was confirmed that the improvement by acipimox on muscle-mitochondrial function was mainly caused by the increase of NAD^+^ level (106), which further indicated that NAD^+^ is also an important participant in myocardial and skeletal muscle aging remodeling.

Based on the important role of mitochondrial function in cellular senescence, the development of mitochondria-targeted approaches to ameliorate cellular senescence is gradually gaining attention. A recent study found that when mitochondria with normal function in the liver of young mice were injected into aged mice via the tail vein, these mitochondria could enter and stably exist in the brain, skeletal muscle, liver, kidney, heart, and lung tissues of aged mice, and improve the learning, memory, and motor functions of aged mice (107).

### 4.3 Maintain telomere length

Many age-related pathologies and progeria syndromes are characterized by faster-than-normal telomere shortening, suggesting that telomere shortening is the cause of body aging (108). Telomerase can maintain telomere length to a certain extent, but the telomere will undergo damage during each cell division and eventually be exposed at the stained ends, resulting in lasting DNA damage. When telomere length reaches the threshold, cells escape the cell cycle and activate senescence or apoptosis (109). Therefore, maintaining telomere length is an important strategy to combat aging and prolong life. Studies have found that the shortening of telomere length is associated with low selenoprotein levels (110). Additionally, the expression of telomerase activity was low in healthy cells, and the telomeres were significantly prolonged after the administration of sodium nitrite, and the growth state of cells significantly changed (111). In addition to the telomerase-mediated telomere maintenance mechanism, there is an alternative lengthening of telomere (ALT) mechanism (112). To regulate telomere length, telomerase uses RNA templates to add telomere repeats to the 3’-end of chromosomes. Except for stem cells, germ line cells, and most tumor cells, most human cells no longer show telomerase activity, mainly due to inhibition of the TERT gene expression (113). Moreover, 15% of telomere length is maintained by ALT mechanisms based on homologous recombination (HR) dependent exchange and/or HR-dependent telomere DNA synthesis (114). Targeted telomere maintenance is, therefore, an opportunity to treat most cancers. Inhibition of tumor cell proliferation by targeting telomerase is an effective way to treat cancer. However, the cytotoxic effect was not shown until the telomere length was shortened to a critical value in the late inhibition of telomerase activity (115). Therefore, effectively targeting telomerase to maintain telomere stability is very important in anti-cell aging and anti-tumorigenesis. Telomere synthesis can be blocked by oligonucleotides that inhibit human telomerase RNA (hTR) function by targeting the template region of hTR. When combined, the oligonucleotides can effectively inhibit the catalytic effect of telomere repeated addition (116). Nucleoside analogs are covalently bound to telomere ends by telomerase and prevent further nucleotide addition due to the lack of the 3’-OH functional groups. Analogs previously used to inhibit human immunodeficiency virus (HIV) reverse transcriptase have also been found to inhibit telomerase reverse transcriptase (hTERT) catalytic sites. Nucleoside analogs studied in telomerase-positive cancers include azidothymidine (AZT), 6-thio-dG, and 5-MeCITP (117). Additionally, hTERT small molecule inhibitors, hTERT-specific immunotherapy, the G4 stable ligand, and therapeutic strategies targeting ALT mechanisms have all been discussed in relevant reviews (118).

Studies have initiated an *in vitro* immune response of T lymphocytes to microorganisms (foreign infections) and found that they observed a telomere transfer response between two types of leukocytes in extracellular vesicles (small particles that facilitate cell-to-cell communication), with T cell telomeres lengthening by 3 kb. After telomere transfer, the recipient T cells became longer-lived and possessed long-term immune memory and stem cell attributes, allowing the T cells to provide long-term protection to the host against lethal infections. This mechanism of telomere recombination fuses telomeres to the ends of T-cell chromosomes, lengthening them by an average of approximately 3,000 base pairs, which is more than 30 times the length of telomerase lengthening. Transferring telomeres protects recipient T cells from replicative senescence and has long-term immune memory and stem cell properties, enabling T cells to provide long-term protection against lethal infections. The telomere transfer reaction between immune cells suggests that cells are capable of exchanging telomeres as a way of regulating chromosome length before the onset of telomerase action. Suggesting that we can slow or cure aging simply by transferring telomeres (119).

### 4.4 Anti-inflammatory therapy

The immune system and inflammation interact during various disease development. An adapted immune system plays an important role in preventing and monitoring the occurrence and progression of tumors, while inflammation promotes the occurrence of tumors and the progression of malignant tumors. Acute inflammation is a stress response to danger signals, cell tissue destruction, and infection in the body. Stopping the occurrence and development of acute inflammation can promote the recovery of body tissues to a certain extent, but when acute inflammation is not controlled effectively and timely, it develops into chronic inflammation and increases cancer risk (120). Some of these drugs have been shown to reduce the risk of developing tumors (121–124). Cytokines, chemokines, and growth factors produced in the TME promote the proliferation, evolution, and growth of cancer cells, while tumor vascularization and immune dysregulation persist, leading to tumor progression, invasion, metastasis, and therapeutic resistance. Therefore, anti-inflammatory drugs alone or combined with immunosuppressive agents can be used as an effective way to inhibit tumor development. The suppression of glycolysis in immune cells by immune checkpoints leads to the suppression of T cell function; therefore, immune checkpoint blockers are used to regain glucose uptake by T cells (125). However, due to the existence of pro-inflammatory and immunosuppressive TME, immunosuppressive drugs are resistant to some extent. Therefore, anti-inflammatory drugs targeting immunosuppressive cells and cytokines can cause immune-mediated rejection of cancer, which has led to the development of an emerging anti-inflammatory, immune therapy to prevent the development of tumors. In addition to this, the powerful role of neutrophils in combating microorganisms can cause tissue damage, and neutrophil-driven inflammation is a unifying mechanism in many diseases, and neutrophils are known to promote angiogenesis and distant tumor metastasis. Targeted granu1ocyte colony-stimulating factors (G-CSF) can inhibit the recruitment of neutrophils and enhance the efficacy of antiangiogenic agents (126). Some viral infections are known to promote tumor occurrence and metastasis; hepatitis B virus (HBV) or hepatitis C virus (HCV) plays a major role in the occurrence of hepatocellular carcinoma (HCC). Meanwhile, HBV and HCV can also lead to cancer-promoting inflammation (127). Some immunosuppressive factors, such as IL-10, is considered immunosuppressive cytokine that promotes tumor cell proliferation and metastasis and can promote tumor progression by recognizing IL-10R on the surface of CD8^+^T cells and inhibiting T cell function (128). A study of routine IL-10 exposure to acute otitis media (AOM) found that tumor diversity was strongly associated with colitis. The intestinal tissue of IL-10 mice treated with aseptic AOM was normal without a tumor. In other words, bacteria-induced inflammation promotes the progression of adenoma to aggressive cancer (129), thus modulating the intestinal flora to target immunosuppressor factors to improve the immune microenvironment. Eradication of *Helicobacter pylori* (HP) in gastric cancer with broad-spectrum antibiotics can prevent gastric cancer in patients with an asymptomatic infection or without precancerous lesions and reduce the development rate of heterotropic gastric cancer in patients with early gastric cancer or high-grade adenoma (130). In addition to antibacterial and antiviral therapy, targeted anti-inflammatory drugs, inhibition of TGFβ signal, cytokines targeting mediated TAMs and MDSCs, and natural anti-inflammatory supplements were all discussed in the relevant review (131).

## 5 Conclusions

Currently, immunotherapies, such as checkpoint blocking and CAR-T cell infusion, have been preferred by an increasing number of patients with cancer and have received satisfactory results. However, the overall immune benefit rate was still limited and varied among tumor types (132). Studies have shown that T cell senescence is an important indicator of cell dysfunction and a common feature in TME. Therefore, preventing and reversing T cell senescence and restoring the effector T cell state is promising for tumor immunotherapy. Currently, MAPK and STAT3 signaling pathways are key targets for controlling T cell senescence (54). Additionally, TLR8 signaling can reverse the inhibition of Tregs and tumor cells and prevent T cell senescence. Therefore, inhibiting the activation of MAPK and TLR8 signal can effectively control T cell senescence and improve the efficacy of immunotherapy. Recently, increasing evidence shows that the immune system plays a double-edged role in tumor occurrence, immune surveillance (133),, or promotion of tumor development (134). The changes in T cell functional states and subpopulations during tumorigenesis are dynamic, and more evidence is needed to demonstrate the changes and plasticity of different T cell states during immunoediting, including senescence and/or exhausted T cells, which are essential for the development of new therapeutic approaches (133). In addition to the mechanism of targeting CD8^+^ T cells in T cell reversal therapy, the targeted therapy of CD4^+^ T cells still needs further research. CD8^+^T cells are prone to senescence, and CD4^+^T cells cannot avoid the fate of senescence. However, there is still a lack of definition, epidemiology, and mechanisms of CD4^+^ T cell senescence, and immune dysfunction associated with CD4^+^ T cell disease states, including cancer remain poorly understood. Targeting related helper cells can aid in understanding the depth of immunotherapy and provide new therapeutic ideas for improving anti-tumor immunity.

NK cells play an important role in considerably improving T cell senescence (135). NK cells, characterized by the expression of CD16 and CD56, are the first line of defense against viral infection and cancer cells (136). Experiments have shown that the population of aging T cells, PD-1^+^, and TIM-3^+^ T cells are also notably reduced after the infusion of amplified NK cells into a healthy body. SASP-related factors, IL-6, IL-8, IL-1a, IL-17, MIP-1a, MIP-1β, and MMP1, decreased considerably, while T cells showed increased cytotoxicity (136). However, until now, the mechanism of NK cells that affect T cell aging and failure remains unclear. Therefore, further studies of these aging and failing T cell populations, their origin, and their function in immunopathological conditions will significantly promote the clinical application of NK immunotherapy. A comprehensive score has been developed to quantify senescent cell load, which helps to indicate the senescent cell load in cancer and its vicinity to facilitate clinical trials of interventions to eradicate TIS/dormant cancer cells and prevent recurrence and metastasis (137).

Further studies are required on the mechanism of T cell aging, and there are still many limitations in experimental transformation. Bioinformatics and single-cell sequencing are becoming important in cancer research. In the era of precision therapy, it is necessary to use relevant analytical techniques for accurate subgroup and marker analysis of T cell senescence. Major clinical challenges include evaluation criteria for the degree of T cell senescence in malignant tumors, application indicators of immunotherapy, and the risk of over-immunization. It is necessary to establish relevant markers and more accurate targets to improve the efficacy of clinical immunotherapy and reduce the damage caused by over-immunity.

## Author contributions

MH: Writing – original draft. YW: Writing – original draft. LF: Writing – original draft. CL: Writing – original draft. FF: Writing – original draft. LL: Writing – review & editing. CS: Writing – review & editing.
